# Human papillomavirus spectrum of HPV-infected women in Nigeria: an analysis by next-generation sequencing and type-specific PCR

**DOI:** 10.1186/s12985-023-02106-y

**Published:** 2023-07-11

**Authors:** Ngozi Dom-Chima, Yakubu Abubakar Ajang, Chinyere Ifeoma Dom-Chima, Esther Biswas-Fiss, Maryam Aminu, Subhasis B. Biswas

**Affiliations:** 1 Department of Medical and Molecular Science, College of Health Sciences, University of Delaware, Newark, DE 19716 USA; 2 Department of Microbiology, Ahmadu Bello University, Zaria, Nigeria; 3 Department of Gynecology, Federal Medical Center, Owerri, Imo State Nigeria

**Keywords:** HPV types, Spectrum, Nigeria, Next-generation sequencing, Polymerase chain reaction

## Abstract

**Background:**

Human papillomavirus (HPV) infection and cervical cancer are leading health problems and causes of death in many parts of the world. There are ~ 200 HPV types that can infect humans. This study aims to understand the spectrum of HPV infections in Nigerian women with normal or abnormal cytology.

**Methods:**

We screened cervical samples from 90 women with possible HPV infections collected in two regional hospitals in Nigeria. The first screening was done using next-generation DNA sequencing (NGS), identifying multiple HPV types in many samples. Thereafter, type-specific PCR analysis was used to verify the NGS-identified HPV types in each sample.

**Results:**

NGS analysis of the 90 samples from the Nigerian cohort identified 44 HPV types. The type-specific PCR confirmed 25 HPV types out of the 44 HPV types detected by NGS, and ~ 10 of these types were the most prevalent. The top five prevalent types found in the Nigerian cohort were HPV71 (17%), HPV82 (15%), HPV16 (16%), HPV6 (10%), and HPV20 (7%). Among the PCR-confirmed HPV types, we found 40.98% high-risk HPV types, 27.22% low-risk HPV types, and 31.15% undetermined HPV types. Among these 25 HPV types in Nigeria, only six were included in the current nine-valent HPV vaccine. We also observed strikingly high multiple HPV infections in most patients, with as many as nine HPV types in a few single samples.

**Conclusions:**

Our NGS-PCR approach of HPV typing in the Nigerian cohort samples unveiled all possible HPV types currently circulating in Nigerian people. We confirmed 25 HPV types using NGS and PCR, with many samples infected with multiple HPV types. However, only six of these types are part of the nine-valent HPV vaccines indicating the need to develop region-specific selective vaccines.

**Supplementary Information:**

The online version contains supplementary material available at 10.1186/s12985-023-02106-y.

## Introduction

Human Papillomavirus (HPV) belongs to the Papillomaviridae family that has five major phylogenetic genera; Alpha (α)-, beta (β)-, gamma (γ)-, mu (μ)-, and nu (ν)-papillomaviruses [1]. Alpha papillomavirus genus is a unique pathogenic HPV type responsible for genital HPV infections. HPV infection is the most common sexually transmitted infection (STI) observed in sites like the lower genital tract, head & neck, and esophagus [1]. The HPV types that cause infection are classified as low-risk HPV (Lr-HPV) and high-risk HPV (Hr-HPV) types [2]. According to the International Agency for Research on Cancer (IARC) 2007 report, low-risk HPV types (2, 3, 6, 7, 10, 11,13, 27, 28, 29, 32, 40, 42, 43,44, 54, 57, 61, 62, 71, 72, 74, 78, 81, 83, 84, 86, 87, 89, 90, 91, and 94) are associated with anogenital warts and benign epithelial lesions, while high-risk HPV types (16, 18, 26, 30, 31, 33, 35, 39, 45, 51, 52, 53, 56, 58, 59, 66, 67, 68, 69, 70, 73, 82, and 85) are associated with cancer [3]. In addition, HPV 77, 97, 102, 106, 114, 117, 125, 160, and 117 have been classified as HPV of undetermined risk (Ur) because of limited data confirming the carcinogenicity of these HPV types [4].

Cervical cancer is the third most common cancer among women, associated with infection with oncogenic HPV types. Cervical cancer accounts for the second most frequent cause of cancer-related death globally. In developing countries, cervical cancer cases are women's leading cause of cancer-related deaths [5]. There were 34.8 new cases and 22.5 deaths per 100,000 women from cervical cancer [6]. In Nigeria, cervical cancer is currently the second most common cancer in women, with about 12,075 new cases diagnosed annually and 7968 deaths [7]. Sub-Saharan Africa has the highest rates of cervical cancer in the world, and it is the most common cause of death among women in 21 of the 48 countries in sub-Saharan Africa [5].

Prophylactic vaccine targeted toward prevalent HPV types has been developed and used in many countries. But research shows that HPV-type prevalence can be geographically specific [2–4]. HPV vaccines (Cervarix-2, Gardasil-4, and Gardasil-9) protect only against types 6, 11, 16, 18, 31, 33, 45, 52, and 58 HPV types [8]. However, despite recent progress, studies on the geographic or regional distribution of HPV infections remain limited in many countries, including Africa. As a result, there is no comprehensive data on HPV genotypes or their geographical distribution in various parts of the world. Given the specificity of strains targeted by a given vaccine, it is essential to determine the spectrum of HPV types in regions with high cervical cancer rates so that region-specific vaccines can be designed.

The HPV type prevalence varies between populations, as it can be country/region specific [9]. Although HPV 6, 11, 16, and 18 are generally believed to be the most common HPV types worldwide [5–7], however, recent data show otherwise. For example, high-risk HPV35 is the most prevalent, while HPV53 and HPV66 are the most prevalent low-risk types in Zimbabwe [10–13]. In South Korea, high-risk HPV 52, 53, and 58 and low-risk HPV42, 44, and 54 are prevalent [10]. Studies in the past predominantly used PCR-based techniques as the screening tool, even though these techniques are limited in the detection range [14–16]. Thus, these techniques can lead to an underestimation of HPV types. In addition, increased PCR cycles can interfere with primer heterogeneity and prevent detecting many HPV DNA types and variants [17]. Hence, it is essential to estimate the prevalence and distribution of HPV types with Next-generation sequencing (NGS), which is comprehensive in HPV type detection and does not suffer from these limitations.

Next-Generation Sequencing is a powerful tool that has expanded our ability to investigate genomic variations and large-scale HPV DNA genotyping. Due to this approach, the NGS-based technique can identify every type of HPV. It is particularly suitable for the HPV types that other methods could not detect [18]. It should be noted that there are around 200 different HPV types worldwide, and these genomes are highly homologous, which could result in overestimating HPV types. Thus, we reanalyzed cervical samples using type-specific PCR, guided by the NGS data, and confirmed the HPV types detected by NGS. In addition, we performed multiple DNA sequence alignments of prevalent HPV types found in the cohort to examine the evolutionary pattern of the HPV types. This study will help model the HPV types needed to produce region-specific HPV vaccines soon. We hope that this study will contribute to preventing and eliminating cervical cancer cases in Nigeria.

## Material and methods

### Participants

The clinics in two regions, Plateau and Imo states, of Nigeria recruited study participants on a rolling basis, and cervical samples were collected from unvaccinated (HPV vaccine) individuals. A total of 90 cases were evaluated. Inclusion criteria for the participants were: 1) age > 18 years, 2) consenting sexually active and non-sexually active women, and 3) with or without cervical cancer. Participants were excluded if they were < 18 years and did not consent. The Ethics Committees of Plateau State Specialist Hospital (PSSH) of Ahmado Bello University, Jos, Plateau State, and Federal Medical Center (FMC), Imo State, approved the research protocol, and the participants signed the informed consent.

### Sample collection and management

Cytobrush was used to collect the cervical samples. The brush was placed in 1 ml of PreservCyt solution and vortex vigorously for 1 min. After overnight incubation at room temperature, the brushes were removed, and ~ 500 µL of the cell suspension was applied to a Whatman FTA (indicating) card (GE Healthcare Life Sciences, Piscataway-away, NJ) and placed at room temperature to air dry. The FTA card is a filter paper product by GE Health Care that stabilizes nucleic acids on contact for long-term storage and transport at room temperature; the color indicator changes from pink to white when the sample is applied successfully.

### Sample processing, rolling circle genome amplification, and next-generation sequencing

Circular DNA of the episomal HPV genome(s) in the cervical samples was amplified by rolling circle amplification (RCA) using the Illustra TempliPhi Amplification kit (GE Healthcare Life Sciences, Piscataway-away, NJ) according to the manufacturer's recommendations. The DNA was eluted from the FTA cards with the organic method following the manufacturer's suggested protocol and stored at − 20 °C until further use. Fifty ng DNA was used to prepare and index each DNA library with the Nextera DNA CD Indexes Sample Preparation kit (Illumina Inc., San Diego, CA). After the library preparation, sample libraries were pooled and quantified using the Qubit fluorometer (Invitrogen). Twenty microliters of the pooled 200-pmol/L libraries were loaded onto the flow cell. Sequencing was performed with iSeq 100 (2 × 150-bp reads paired-end).

### NGS data analysis

FASTQ Reads generated from NGS were evaluated for quality and quantity with BaseSpace software (Illumina). The average quality of reads from each sample was 37 Phred scores, and the length was 200 bp before trimming. Trimming and FASTA conversion were performed with Bugaco fastq to fasta converter (FASTQ to Fasta Sequence converter) to remove base calls with Phred quality and convert fastq files to fasta files as PAVE database only maps fasta files. Converted fasta files from each sample were mapped against a set of reference sequences from over 200 HPV types available at the PAVE database (http://pave.niaid.nih.gov). HPV was defined by at least one read aligned to the L1 ORF (the most conserved and used to classify HPV types) of a given HPV reference PV-specific blast (http://pave.niaid.nih.gov).

### HPV DNA typing with type-specific (ts-PCR) primers

Based on the NGS data, all 90 samples were tested by PCR analysis using HPV-specific primers to verify the identified HPV types in the NGS analysis. The following HPV types were examined by ts-PCR: 6, 7, 11, 14, 16, 18, 19, 20, 21, 25, 31, 32, 34, 39, 43, 45, 58, 59, 61, 66, 68, 70, 71, 73, 82, 84, 85, 86, 87, 90, 91, 92, 97, 114, 118, 152, 195, 196, 220, and 224 using type-specific oligonucleotide primers as described (Additional file 1: Table S1). In addition, NGS-identified HPV type(s) in each patient sample was analyzed by ts-PCR using a single PCR run for each type. The ts-PCR analysis was necessary as NGS analysis indicated the presence of multiple HPV types in many cervical samples to eliminate putative false positives. Primer sequences and fragment sizes are shown. In Additional file 1: Table S1. The PCR was performed in a final reaction volume of 25 µL, containing 10.5 µL of water, 1 µL of DNA at 10 ng/µL, 1 µL of each primer at 10 µM, and 12.5 µL Taq 2X Master Mix (New England Labs Inc). The Taq 2X Master mix consisted of 10 mM Tris–HCl, 50 mM KCl, 1.5 mM MgCl2, 0.2 mM dNTPs, 5% Glycerol, 0.08% IGEPAL^®^ CA-630, 0.05% Tween^®^ 25 units/ml, Taq DNA Polymerase. The PGEM DNA was used as a positive control, while a No Template Control (NTC) served as a negative control to validate the PCR reaction efficiency. Amplification conditions were as follows: 40 cycles of 95 °C for 1 min, 55 °C for 1 min, and 72 °C for 2 min, and a final extension at 72 °C for 7 min. The PCR products were electrophoresed using a 2% agarose gel in 1 × TAE buffer, stained with gel red (100,000×), and photographed under UV illumination. Intra-assay variability was determined in triplicate in three experiment runs.

### Phylogenetic analysis

We performed a phylogenetic analysis to examine the evolution of select papillomavirus. Complete DNA sequences were obtained from PAVE for HPV type 3, 6, 7, 14, 16, 18, 20, 21, 32, 33, 39, 43, 52, 58, 59, 66, 71, 82, 85, 90, 91, 114, 118, 196, and 152. The HPV DNAs were subjected to multiple alignments using MEGA software [19]. The multiple sequence alignments led to the phylogenetic trees using the Maximum Likelihood using the Kimura 2-Parameter model.

### Data analysis

The mean, standard deviation, and coefficient of variation (CV) were calculated for all observed frequencies of High risk, low-risk, and Undetermined-risk HPV type-specific infections detected by NGS and verified by TS-PCR. All data analysis and graphs were performed using GraphPad Prism 5 (GraphPad Software) and Microsoft Excel software.

## Results

**Fig. 1:**
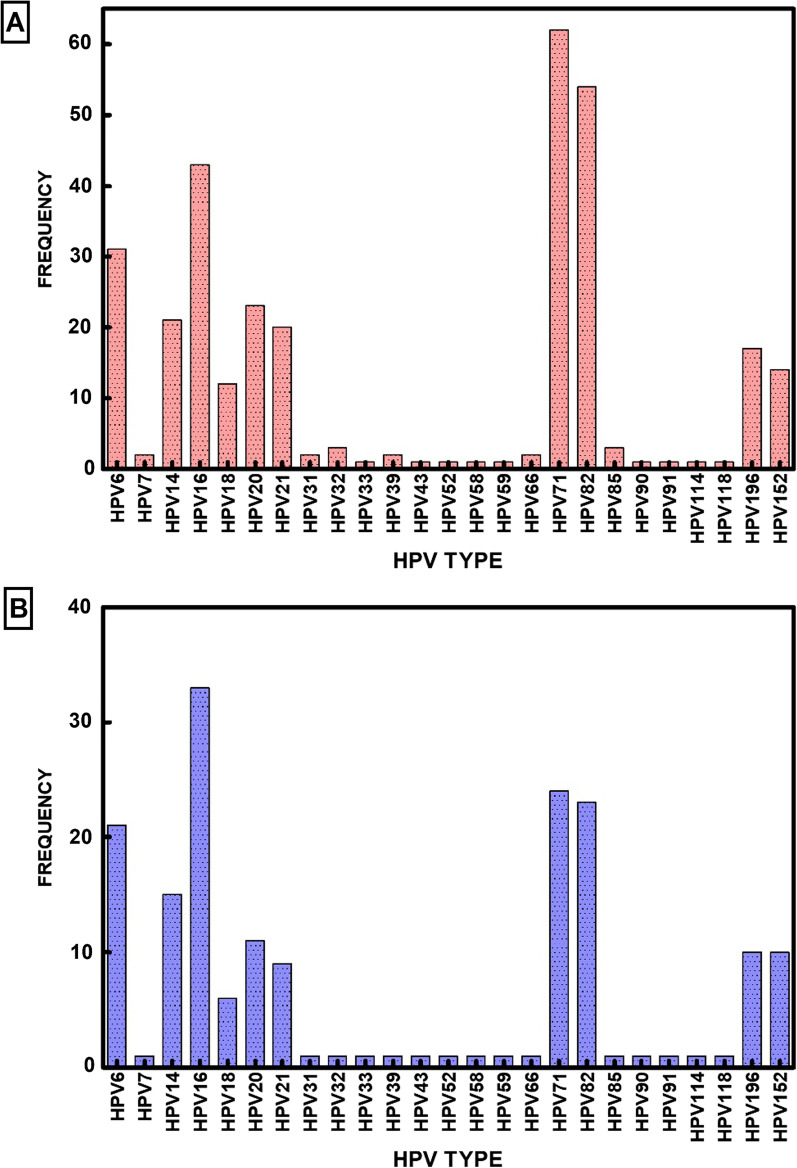
Prevalent HPV types by next-generation sequencing and type-specific PCR **A** Frequency of HPV types detected by next-generation sequencing. **B** Frequency of HPV types detected by type-specific PCR. Bars indicate the percentages of HPV types

Our goal in this study was to determine the diversity and prevalence of HPV types in Nigeria using DNA amplification and sequencing. The samples were collected and transported using a simplified FTA card protocol (GE Life Sciences, Piscataway, NJ) methodology. RCA amplified the DNA samples. The amplified DNA was analyzed by NGS sequencing and tsPCR. We have analyzed cervical samples from 90 cervical samples from two regions of Nigeria (results are presented in Additional file 2: Table S2). NGS sequencing and tsPCR identified a diverse group of 25 HPV types in these 90 samples (Fig. 1). A critical evaluation of the results showed HPV types 6, 7, 14, 16, 18, 20, 21, 31, 32, 33, 39, 43, 52, 58, 59, 66, 71, 82, 85, 90, 91, 114, 118, 152, and 196. These HPV types should be considered major HPV types prevalent in this population (Table 1).

**Table 1:** Mean, standard deviation (SD), and coefficient of variation (CV) of observed frequencies of High risk, low-risk, and Undetermined-risk HPV type-specific infections detected by NGS and verified by TS-PCR

	HPV TYPE	NGS	TS- PCR	MEAN	SD	CV
Low-risk HPV types	HPV71	62	24	38.6	19	2.03
	HPV6	31	21	25.5	5	5.10
	HPV32	3	1	1.73	1	1.73
	HPV7	2	1	1.41	0.5	2.82
	HPV43	1	1	1	0	0
	HPV90	1	1	1	0	0
	HPV91	1	1	1	0	0
High-risk HPV types	HPV16	43	33	37.7	5	7.53
	HPV82	54	23	35.2	15.5	2.27
	HPV18	12	6	8.49	3	2.83
	HPV85	3	1	1.73	1	1.73
	HPV31	2	1	1.41	0.5	2.83
	HPV39	2	1	1.41	0.5	2.83
	HPV66	2	1	1.41	0.5	2.83
	HPV33	1	1	1	0	0
	HPV52	1	1	1	0	0
	HPV58	1	1	1	0	0
	HPV59	1	1	1	0	0
Undetermined-risk HPV types	HPV14	21	15	17.7	3	5.92
	HPV20	23	11	15.9	6	2.65
	HPV196	17	10	13.0	3.5	3.73
	HPV152	14	10	11.8	2	5.91
	HPV21	20	9	13.4	5.5	2.44
	HPV114	1	1	1	0	0
	HPV118	1	1	1	0	0

### NGS detection and identification of HPV genotypes

NGS was performed to detect HPV types in the cervical samples, and a total of 44 HPV types were identified in this cohort. We identified the following HPVs: 6, 7, 11, 14, 16, 18, 19, 20, 21, 25, 31, 32, 33, 34, 39, 43, 45, 52, 53, 56, 58, 59, 61, 66, 68, 70, 71, 73, 82, 84, 85, 86, 87, 90, 91, 92, 97, 114, 118, 152, 195, 196, 220, and 224. HPV82 was the most detected Hr-HPV type, HPV71 was the most detected Lr-HPV type. This observation indicates that NGS is a highly sensitive method for HPV detection and typing due to its ability to detect multiple HPV-type infections and novel types that may escape other molecular methods. Overall, we found 18 Hr-HPV, 13 Lr-HPV, and 13 Ur-HPV indicating the unbiased detection approach of NGS. In addition, among these 13 Ur-HPV types found, 10 were beta papillomavirus, 2 were gamma papillomavirus, and 1 was alpha papillomavirus. This is consistent with the NGS's ability to detect multiple infections with different families of papillomavirus.

Our results indicate that the distribution of HPV types were as follows: Ur-HPV was higher than Hr-HPV and Lr-HPV with Ur-40%, Hr-34%, and Lr-26% respectively (Table 2). HPV type 16 is the most prevalent strain, followed by types 6 and 18, which are also prevalent to a lesser extent. However, NGS unearthed several lesser-known HPV types, 14, 19, 20, 21, 25, 71, and 82, which appear to be prevalent in the Nigerian population. We did not detect any sample that was devoid of HPV DNA in the cohort of 90 samples.

**Table 2:** Hr-HPV, Lr-HPV, and Ur-HPV positive rates detection rate by NGS and ts-PCR

	Hr-HPV (%)	Lr-HPV (%)	Ur-HPV (%)
NGS	34	26	40
ts-PCR	40	27	33

Comparative analysis between NGS and ts-PCR methods, in detection HPV DNA types in cervical specimens

In the cohort, multiple HPV type infections between 2 and 19 HPV types were detected in 80 samples, while we detected a single HPV type infection with only one HPV type in only ten samples. The overall occurrence of multiple HPV-type infections was high at 87.78% (95% CI 79.43–93.04%). Additionally, a surprising finding of NGS analysis is that 31 of 90 patients appeared to harbor four or more HPV types simultaneously. In contrast, single HPV type infection was low at 12.22% (95% CI 6.96–20.57%).

We calculated the frequency of HPV types to determine the detection rate of HPV risk types in single and multiple infections. In single infections, we found Lr-HPVs were higher than Hr-HPVs with 57.55% compared to 43.45% using NGS detection; 33.33% for Lr-HPVs and 66.67% for Hr-HPVs using NGS + ts-PCR combined detection. In samples with multiple infections, we observed that Ur-HPVs were detected more than Hr-HPVs and Lr-HPVs (44.21% vs. 32.05% and 23.74%) using nGS detection (Table 3). Using NGS + ts-PCR combination, Lr-HPVs were 22.5%, Hr-HPVs were 41.88%, and Ur-HPVs were 35.63% as shown in Table 3.

**Table 3:** Overall detection rate of LR-HPV, Hr-HPV, and Ur-HPV types in women with single or multiple infections by NGS and ts-PCR

	Single infection			Multiple infections		
	Lr-HPV (%)	Hr-HPV (%)	Ur-HPV	Lr-HPV (%)	Hr-HPV (%)	Ur-HPV (%)
NGS	57.55	43.45	0	23.74	32.05	44.21
ts-PCR	33.33	66.67	0	22.50	41.88	35.63

### Type-specific PCR validates HPV genotypes detected

NGS analysis is probably the most sensitive methodology for HPV analysis because of the high sequence homology of HPV types, large diversity of the virus family, and multiple HPV strains infecting a single individual. However, we could not disregard the possibility of false positives even for NGS. Therefore, we decided to examine each HPV type detected by NGS in each sample by single ts-PCR analysis. Thus, the NGS data of HPV types were critically re-examined using a second methodology to eliminate any potential errors of sequencing as well as any error that could arise due to high sequence homology among various HPV types. Using type-specific PCR (ts-PCR) for each of the 44 HPV types identified by NGS. We confirmed 25 HPV types comprising 11 Hr-HPV, 7 Lr-HPV, and 7 Ur-HPV. In all, we found HPV type 3, 6, 7, 14, 16, 18, 20, 21, 32, 33, 39, 43, 52, 58, 59, 66, 71, 82, 85, 90, 91, 114, 118, 152, and 196 in the sample cohort in this study. The five most frequent HPV genotypes in descending order among the types detected were HPV14, 16, 20, 71, and 82.

To verify the detection rate of ts-PCR to identify the risk types of HPV infection. We found that HPV16 was the most detected Hr-HPV type, HPV71 was the most detected Lr-HPV type, and HPV14 was the most detected Ur-HPV type. Nineteen HPV types from NGS sequencing were not detected by ts-PCR (Table 4). Our results indicate that among the 7 Ur-HPV types detected by ts-PCR, six were beta papillomavirus, and one was alpha papillomavirus. We observed that the detection rate of NGS + ts-PCR was 40% for Hr-HPVs, 27% for UrHPVs, and 33% for LrHPVs (Table 2).

**Table 4:** Detection frequencies of Hr, Lr, and Ur HPV type-specific infections, detected by NGS and not by PCR^†^

HPV TYPE	NGS	Ts-PCR
*HPV19*	25	0
*HPV25*	19	0
*HPV195*	18	0
**HPV56**	4	0
**HPV53**	3	0
HPV11	2	0
**HPV34**	2	0
**HPV68**	2	0
HPV84	2	0
HPV86	2	0
HPV97	2	0
**HPV45**	1	0
**HPV61**	1	0
HPV70	1	0
**HPV73**	1	0
HPV87	1	0
*HPV92*	1	0
*HPV220*	1	0
*HPV224*	1	0

^**†**^Non-bold face indicates Low-risk type; boldface indicates High-risk types; *italicized* indicates Undetermined risk type

The overall prevalence of single HPV-type infection was low-. To assess the rate of HPV risk types detected in single-type and multiple-type infections, we calculated the frequency of these types. In single infections, we found that the prevalence of HrHPVs was higher than LrHPVs (66.67% vs. 33.33%). Ur-HPVs were not observed in single infections. In multiple infections, we found more HrHPVs than UrHPVs or LrHPVs, with a distribution of 41.88%, 35.63% and 22.50% respectively using NGS + ts-PCR combined detections (Table 3).

### HPV DNA phylogenetic analysis

**Fig. 2:**
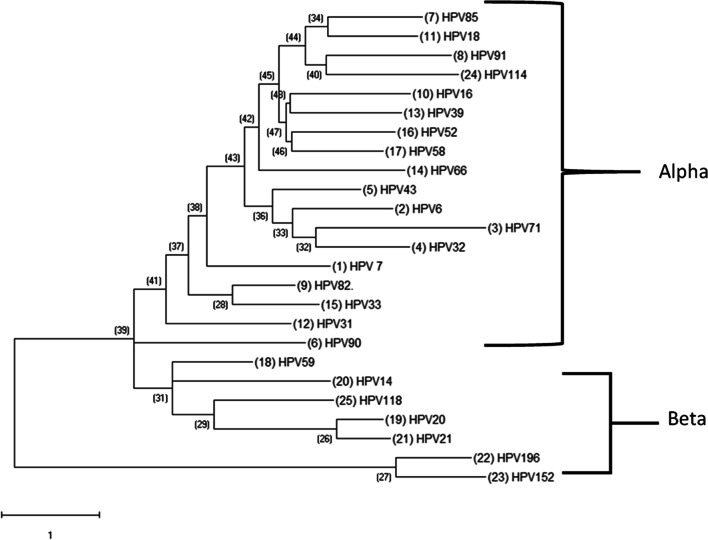
Phylogenetic Analysis of select Papillomavirus found in the cohort. HPV DNA sequences for each papillomavirus were obtained from PAVE and used for MEGA analysis. The resulting multiple alignments were then used to generate a phylogenetic tree to analyze the evolutionary patterns of select papillomavirus. Clades were identified related to the classical Papillomavirus genera indicated on two genera of the PV: alpha-papillomavirus genera and Beta-papillomavirus. Phylogenetic trees were constructed by the Maximum Likelihood method and the Kimura 2-Parameter model by MEGA package

We have examined the evolutionary patterns of selected HPV types and have identified two distinct clade groups: one comprising the α-papillomaviruses and the other comprising the β-papillomaviruses. The α-clade possessed the largest evolutionary diversity compared to the β-clades. This indicates a significant evolutionary divergence of the α- to the β-papillomaviruses. In addition, HPV71 showed a long branch compared to the other members of the α-papillomavirus genus in terms of the number of substitutions per site (Fig. 2).

## Discussion

Human papillomavirus is an infectious and primarily sexually transmitted virus and the leading cause of cervical cancer worldwide [20]. Cervical cancer is a burden most countries in the sub-Saharan Africa and Nigeria is no exception with 763,000 deaths reported in 2019 [5, 21]. HPV vaccination status of women in Nigeria remains unknown. Nigeria's epidemiology of the HPV family of viruses has never been studied extensively. In this report, we have collected cervical samples collaboratively from a cohort of 90 volunteers from two different regions of Nigeria and analyzed the DNA samples for HPV types using NGS. The data from NGS was further examined using ts-PCR analysis in each sample. We report here the data for all HPV types as well as confirmed HPV types found in the cervical samples in this cohort.

**Fig. 3:**
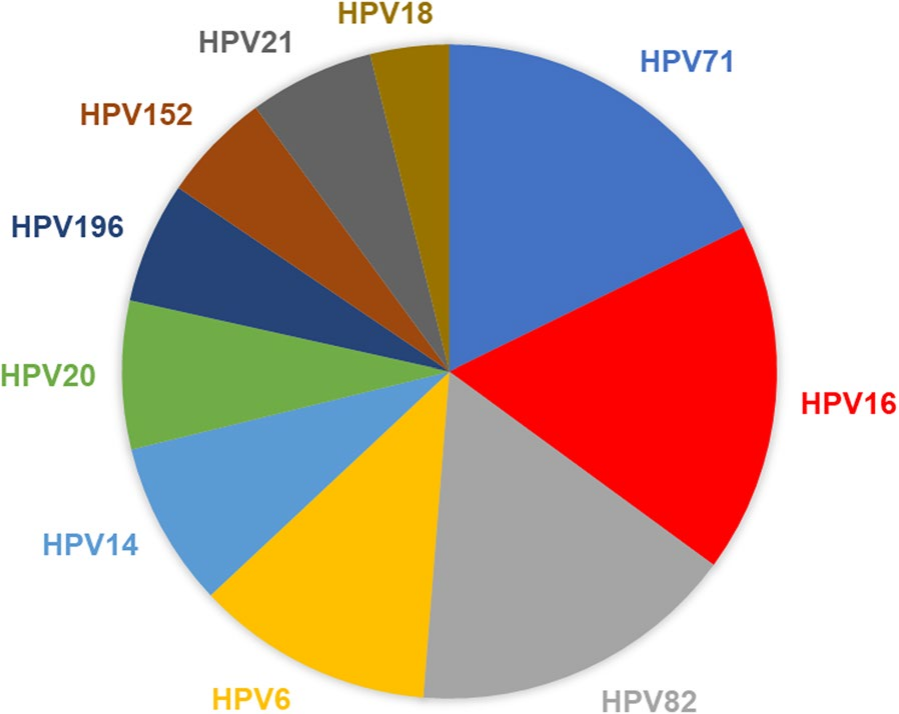
Pie chart representing the top ten prevalent HPV types in Nigeria. Among the HPV types detected, the mean of the top ten detected with NGS and verified by ts-PCR were used to determine the distribution

**Fig. 4:**
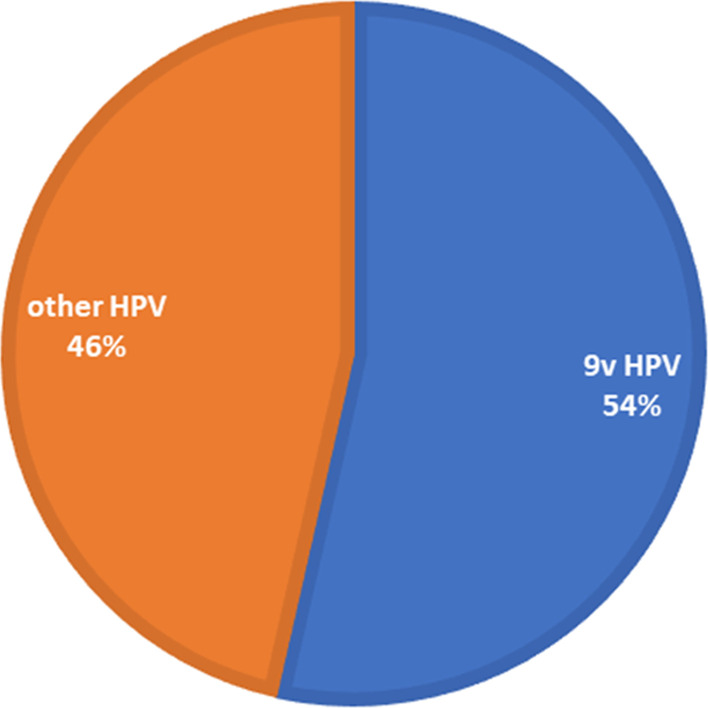
The detection rate of 9v (Gardasil 9) human papillomavirus (HPV). Of the 44 HPV genotypes found in the Nigerian cohort, 63% were covered by the nine-valent Gardasil^®^ 9 HPV vaccine

It is generally assumed that HPV6, HPV11, HPV16, and HPV18 are the most common strains of HPV [8]. However, previous studies indicate that the overall prevalence of HPV types varies globally [22–28]. HPV16 is the most predominant type found in many studies with patient cohorts from other countries like South Korea, Sweden, and Slovenia [29–32]; our results suggest that the spectrum of HPV types found in Nigeria is unique and could be representative for this region of Africa. Our study found HPV types 16, 71, and 82, as the top three prevalent HPV types (Fig. 3). The total number of detected HPV genotypes targeted by the 9v HPV vaccine was 63 (63/117; 53%) (Fig. 4). The HPV types found in this study are like those found in other countries. For example, most common HPV in Thailand, HPV71, was detected in 10.3% of the population [33]. In Japan, 4.8% of the female population had HPV52 [34].

Employing various molecular techniques in past epidemiological studies has revealed the extent of HPV infection burden in different countries [35–38]. We used NGS for unbiased HPV detection and ts-PCR-confirmation to detect HPV typing in this study. NGS identified a total of 44 HPV types in these samples, whereas ts-PCR confirmed 25 of these 44 HPV types. It is difficult to explain why NGS detected 19 HPV types that could not be verified by ts-PCR. It should be noted that HPV types are highly homologous; thus, fortuitous identification of an HPV type as false-positive could not be ruled out. With NGS, we observed 18 Hr-HPV, 13 Lr-HPV, and 13 Ur-HPV, and with ts-PCR, the presence of 11 Hr-HPV, 7 Lr-HPV, and 7 Ur-HPV in this cohort. HPV16 was the most common type, followed by HPV types 14,,20 71, and 82, with ts-PCR. Ur-HPV types were not seen with single infections. In samples with multiple HPV infections, Hr-HPV was detected more than Lr-HPV than Ur-HPV. Ts-PCR detecting and identifying more Hr-HPV is consistent with other studies showing that ts-PCR identified more Hr-HPV, especially HPV16 [39, 40].

In our experiments with NGS, data shows HPV82 as the most detected Hr-HPV type, HPV71 was the most detected Lr-HPV type, and HPV19 was the most detected Ur-HPV type. Ur-HPV was not present in any single HPV infection. Among the confirmed HPV spectrum data, HPV16 was the most predominant. However, HPV6, HPV14, HPV20, HPV71, and HPV82 are well-represented in the HPV spectrum for Nigeria (Fig. 2). Surprisingly, most of the Ur-HPV detected were from beta and gamma papillomavirus families. The five major phylogenetic genera of HPV are alpha (α)-, beta (β)-, gamma (γ)-, mu (μ)-, and nu (ν)-papillomaviruses [41]. Beta and gamma HPV types have been mainly cutaneous, even if detected in other anatomical sites apart from the skin [42, 43]. However, there is limited knowledge about the role of beta and gamma HPV infection in carcinogenicity in the cervix.

Phylogenetically, the results show an alpha and beta papillomavirus genus. There was a significant evolutionary divergence of the beta papillomaviruses from the root of the cladogram. HPV71's long branch seems to have diverged more than its sister branch, HPV32, and other members of the alpha-papillomavirus genus. This divergence indicates nucleotide substitutions that significantly changed the genetic distance [44]. Interestingly, HPV59, an alpha-papillomaviruses, did not sort out with the other members of the alpha-papillomavirus genus. But it did get classified with the beta-papillomavirus clade.

We believe that using ts-PCR, a standardized method as a confirmatory test, and NGS, a more sensitive molecular assay for HPV detection and typing, did help in reducing possible false positives. In combination, these techniques allow for the accurate determination of single as well as multiple infections of HPV types. The results of our studies apply only to the current infection status. In this study, we could not collect any data on the HIV status of this cohort of patients. Women infected with HIV have a higher prevalence of HPV infection than HIV-negative women HIV as shown in an earlier study from Brazil [45]. Multiple HPV infections commonly occur in HIV-positive immune compromised women and can simultaneously include over 20 HPV types [33, 46, 47]. Comparing our data with the result of HIV-coinfected patients in Brazil and elsewhere makes us wonder whether the cases of multiple HPV-infected Nigerian cohorts were also coinfected with HIV [33, 45–47].

The significance of the presence of multiple HPV types in a single individual in terms of carcinogenesis remains unclear. However, multiple HPV types was discovered by Siqueira et al. in HIV/HPV coinfected pregnant women in Brazil in 2016 [45]. Therefore, these women were likely immune compromised due to HIV infection. In the Nigerian cohort, a limitation of the study is that we did not have data for other health conditions of the volunteers; thus, we could not determine HIV infection. In addition, other health conditions may also lead to a weaker immune system leading to multiple HPV infections. Multiple HPV infections probably enhance the possibility of carcinogenesis. In fact, HIV/HPV coinfected patients are six times more likely to develop cervical cancer than those without HIV [5]. Thus, multiple HPV infection could very well be the best indicator for HPV related cervical cancer prognosis.

In this study, we have detected 18 Hr-HPV, 13 Lr-HPV, and 13 Ur-HPV types in all. The significance of the Hr-HPV and Lr-HPV types has been well-known and discussed in many publications [3]. However, Ur-HPV types remain uncharacterized due to the lack of sufficient clinical data, and their role in the development of cancer remains unknown. In earlier studies, we have identified specific sequence patterns that can be used as fingerprints for carcinogenic or high-risk HPV types [48–50]. In the origin of DNA replication of all HPV types, there are primarily three 12 mer conserved DNA sequences, ACCG-NNNN-CGGT, which are the binding sites for HPV-encoded E2 protein that regulates DNA replication, transcription, and carcinogenesis in HPV infected cells [50]. Yilmaz et al. [48] discovered mutations in these conserved sequences in all known high-risk HPV types. Using Yilmaz classification, based on DNA sequence fingerprints, Ur-HPVs can be classified accordingly [48].

In this study we used 90 samples due to difficulties in procuring samples, the limited number of samples (90 samples plus control samples) that NGS could process in one run. In future studies, we would like to see a larger patient cohort with a detailed health history, such as HIV status, pregnancy, drug use, etc., and more resources. Needless to mention, NGS is the only effective means of comprehensive identification of multiple HPV types in patient samples as we show here. However, NGS alone (and even more with ts-PCR combination) is expensive. It will be difficult to carry out such large-scale HPV NGS studies without substantial funding from an international agency such as the World Health Organization (WHO).

## Conclusions

We have attempted to study the HPV spectrum in Nigeria. Perhaps, this study could be the first such study of HPV in Western Africa. Our results indicated that the spectrum of HPV in Nigeria is unique and distinct from other non-African countries based on the published reports. Therefore, such studies for each country/region could be useful in understanding the distribution of the virus and its systematic elimination. We hope that our study encourages other HPV researchers all over the world to assess HPV spectra in all regions of the world so that a complete global map of HPV type distribution could be assembled. These data will likely be necessary to develop next-generation vaccines against this pervasive virus family.

Even though NGS is a reliable and sensitive methodology, PCR confirmation of NGS data help in identifying true positives. Widespread application of the currently available HPV vaccines in Nigeria could reduce the total number of cases of HPV infections; however, other equally potent HPV types already present in Nigeria could easily replace the vaccine-eliminated types. Thus, a more selective vaccine designed for the region could be more effective for Nigeria.

## Supplementary Information

**Additional file 1**: **Table S1**. HPV type-specific primer sequences and product size.

**Additional file 2**: **Table S2**. HPV types found in the participants.
