# Supplementary material for: Human papillomavirus spectrum of HPV-infected women in Nigeria: an analysis by next-generation sequencing and type-specific PCR
Source: Virol J. 2023 Jul 11;20:144. doi: 10.1186/s12985-023-02106-y (PMC10337082; doi:10.1186/s12985-023-02106-y)
Supplement: Supplementary file 1 — Additional file 1: Table S1. HPV type-specific primer sequences and product size. [file 12985_2023_2106_MOESM1_ESM.pdf]

Supplementary Table S1: HPV type-specific primer sequences and product size.

HPV TYPES	PRIMER SET<br>(5'-3') FORWARD<br>(3'-5') REVERSE	PRODUCT SIZE
HPV6	TCCATTAGCCTCCACGGGTG<br>TAGTGGGCCTATGGCTCGTC	75
HPV7	AGTGTATTTACCACCGCCCA<br>CGGGCAAGCGTACTCTAAAC	206
HPV11	CGAGCAGACGTCCGTCCTCG<br>GGAATACATGCGCCATGTGG	360
HPV16	TCAAAGCCACTGTGTCCTGA<br>CGTGTTCTTGATGATCTGCAA	119
HPV18	CCGAGCACGACAGGAACGACT<br>TCGTTTTCTTCCTCTGAGTCGCTT	173
HPV14	GGATGATGACGAACAGTGGC<br>TGAGTCTGCTTGTCCTCCTG	220
HPV19	AGGAGGGCGAAAATGGAGAA<br>TAACACCCTTTCGCCTAGCA	166
HPV20	GTTGTTGTTGCCAGCTACCA<br>CCTTCATCAGCGCTGTCTTC	209
HPV21	AAAGTCCAAGCGGTGTCAAC<br>TGAGTCTGCTTGTCCTCCTG	197
HPV25	TGTTAGGTCAGCATTCGGGT<br>GTTTCAACACGAGGCACAGT	223
HPV31	CTACAGTAAGCATTGTGCTATGC<br>ACGTAATGGAGAGGTTGCAATAACCC	155
HPV32	GGACATTTCTCCTCCCACCA<br>CATAGTAGGGCGAGGTCCAG	244
HPV33	GCACACTCCATGCGTATCAG<br>ATGATAGATGATGTAACGCC	456
HPV34	GGAGACACTGTGCAAACGTT<br>TTGCAGTCCCATTTCACGTG	154
HPV39	CACAGAGTCACAACGCCAAA<br>AAAGCGGGACATTGCAACAT	199
HPV43	CTAAGTCCCAGGTTGCATGC<br>CCCCTCCCACACCCTTTATT	188

HPV45	ACCAGATTTGTGCACAGAAT<br>TTTTTTCCAGTGTCTCTCCA	236
HPV52	TTCAGAGTGTTGGAGACCCC<br>TGTGGCTTGTTCTGCTTGTC	197
HPV53	GGTGGTGCCCTGTTTACAAG<br>TGTCCTTGCTGCCATCAAAC	209
HPV56	CAGGATTTGGCGCTATGGAC<br>CCCCAACTTTACCAGCCCTA	198
HPV58	TGCTGTAGAGGACTGTGTGG<br>CATTTAAGTCTGCGTCGCCA	188
HPV59	ATGTGCAGTACCAGTGACGA<br>GTAGACTGAGGGTGCTGTGT	173
HPV61	CACGACAAGCAGGACTAACG<br>ATCGTTGTGGCTGCGTATTC	189
HPV66	GGGTTTGGTGCAATGGACTT<br>CCCCAACATTACCTGCCCTA	196
HPV68	TTTTGTATGGCCGGGTTGTG<br>AGGAGATACGTTGGTTGCCA	212
HPV70	TGTAACCCGCCCTTCATCTT<br>TGAACCTGTGCCCCAATTTG	213
HPV71	CCCGTCTGGCTGATGTAGAT<br>TTCGCCAATCCCCTTCTTCT	183
HPV73	AGACACACATCCTGGTCCAG<br>CTTTGCATCAGTAGGTCGCC	203
HPV82	TTGCAGGTGTTCGAGTGTTG<br>GCCTCCACATAAAACCACCC	191
HPV84	GGGAGGTGTATATGGGAGGC<br>CCGGTGTCCTTGTGTAGAGT	245
HPV85	TACGAGATCTCCAGCAGCTG<br>GTCCTCGTCCTCATCCTCTG	194
HPV86	ATTCACCATGCATGCGACTC<br>GAATGCTCGCTTGTCTCCTG	213
HPV87	GAGTACTCCAGCTACGGACC<br>CAGTTGCTGCACTTTCCTGT	179
HPV90	CATCGCTCTAAACGTCGCAA<br>AGAAGACACCCAAGCTACCC	151
HPV91	GTTGTGGGCTCAAACGTCAT<br>GGCTCTCCTCCAACAGTTCT	230

HPV92	GAAGAAACGAAAACGGGCCT<br>GCAGGCGATCACTATTTGCA	168
HPV97	GCAAAACAACCTTGGAACGC<br>TTCAACGGTTTCTGGCACTG	235
HPV114	TGGCACGTGTTACCATACCT<br>GCAGCAACACACTACACCAA	204
HPV118	TGCGAGCCAGGAGAGTTAAA<br>CACACGTACTCCAGGACCTT	236
HPV152	AACAGGAGAGTGAGGACAGC<br>AGGCGGAGGAGTATTGTCAG	242
HPV195	TATGGGAGTGCTGGTGTGTT<br>ATGTCTGCAGGCCCAATAGT	170
HPV196	TTAGCTCCACTGGACACTGG<br>TTGTTGCTTCTCGGTGGTTG	182
HPV220	CCTTTACCTGCCTCCTCCAA<br>CCTTATCACCCGCCACATTG	152
HPV224	TGGTACAGGGAGCAGTCTTG<br>GCCCCTGGACGAACAATTTT	201
