# Supplementary material for: Human papillomavirus spectrum of HPV-infected women in Nigeria: an analysis by next-generation sequencing and type-specific PCR
Source: Virol J. 2023 Jul 11;20:144. doi: 10.1186/s12985-023-02106-y (PMC10337082; doi:10.1186/s12985-023-02106-y)
Supplement: Supplementary file 2 — Additional file 2: Table S2. HPV types found in the participants. [file 12985_2023_2106_MOESM2_ESM.pdf]

Supplementary Table S2: HPV types found in the participants[†]

Participant's ID	NGS	Ts-PCR
Patient #1	6, **18**	**18**
Patient #2	6, **18**	**18**
Patient #3	6	6
Patient #4	6, **16, 18**	**16**
Patient #5	6, **16, 18**	6, **18**
Patient #6	6, **16, 18**	6, **18**
Patient #7	6	6
Patient #8	6, **16, 18**	6, **16, 18**
Patient #9	6, **16, 18**	**16**
Patient #10	6, **16, 18**	6**, 18**
Patient #11	71, **82**	None
Patient #12	6, 71, **82**	6
Patient #13	71, **82**	None
Patient #14	6	6
Patient #15	*14, 19, 20, 21, 25*, 71, **82**, *195, 196*	*14, 20, 21*
Patient #16	**16, 18, 32**, **34, 85,** *92*	**16, 32,** *92*
Patient #17	6	6
Patient #18	**16**	**16**
Patient #19	6, **16**	6, **16**
Patient #20	6, 71, **82**	6
Patient #21	*14, 19, 20, 21, 25*, 71, **82**, *152,195, 196*	*14, 21,* 71, **82**, *196*
Patient #22	71, **82**	71, **82**
Patient #23	6, 71, **82**	6, 71, **82**
Patient #24	6, *14, 19, 20, 21, 25*, 71, **82**, *152,195, 196*	21, 71, **82, 152**
Patient #25	6, **16, 18, 31, 45,** 71, **82**, **85,** *97*	6, **31, 45,** 71, **82,85**
Patient #26	71	71
Patient #27	6, *14, 19, 20, 21, 25*, **66,** 71, **82**,*196*	6, *14, 20, 21,* **66,** 71
Patient #28	71, **82**	71
Patient #29	6, *14, 19, 20, 21, 25*, 71, **82**, *118, 152,195, 196*	6, *14, 20, 21,* 71, **82**, *118, 152, 196*
Patient #30	6, *14, 19, 20, 21, 25*, 71, **82**, *118, 152,195, 196*	6, *14, 20, 21*, 71, *152, 196*
Patient #31	71, **82**	71, **82**
Patient #32	6, *14, 19, 20, 21, 25*,**53**,71, **82**, *195*	6, *14, 20, 21*,71, **82**
Patient #33	*14, 19, 20, 21, 25*, **18**, 71, **82**, *152, 196*	*14, 20, 21,* 71, **82**, *152*
Patient #34	11, **16**, 71, **82**	71, **82**

Patient #35	*14, 19, 20, 21, 25*, 71, **82**, *152,195, 196*	*20, 21,* **82**, *152,196*
Patient #36	6, **18,** *14, 19, 20, 21, 25,* 71, **82**, *152,195, 196*	6,*14, 20, 21,* 71**, 82,** *152*
Patient #37	18	None
Patient #38	*14, 19, 20, 21, 25,* 71, **82**, *195, 196*	71, **82**, *196*
Patient #39	71	71
Patient #40	**16,** *19, 20,* 71, **82**	**16, 82**
Patient #41	6,*14, 19, 20, 21, 25*, 71, **82**, *152,195, 196*	*14, 20***, 82,** *152*
Patient #42	*20,* 32, 71, **82**	*20,* **82**
Patient #43	**16, 18**	None
Patient #44	*14, 19, 20, 21, 25,* 71, **82***, 152,195, 196*	*14, 196*
Patient #45	71, **82**	71, **82**
Patient #46	6, 71, **82**	None
Patient #47	6, 71, **82**	6
Patient #48	**14, 16,** *19, 20, 21, 25*, 71, **82**, *152,195, 196*	**16**
Patient #49	**16, 53**	**16**
Patient #50	**16,** 71	**16**
Patient #51	**16**, 71, **82**	**16**, 71, **82**
Patient #52	**16**, *19,* 71, **82**	**16**
Patient #53	*14,* **16,** *19, 20, 21, 25,* 71, **82**, *152,195, 196*	**14, 16,** *20, 152, 196*
Patient #54	*14,* **16,** *19, 20, 21, 25*,**31,** 71, **82**, *152,195, 196*	**14, 16,** 71, *152, 196*
Patient #55	**16,** 71	**16**, 71
Patient #56	**16**, 71, **82**	None
Patient #57	**14, 16,** *19, 20, 21, 25,* 71, **82**, *152,195, 196*	**16,** *14*
Patient #58	6, 11, **16, 31, 45, 68, 73, 85,** *97*,**7, 33, 39, 52, 58, 59,** 84, 86, *220, 224*	6, **31, 33, 39, 52, 58, 59**
Patient #59	**16**, 71, **82**	**16, 82**
Patient #60	**16**, *97*	None
Patient #61	**14, 16,** *19, 20, 21, 25,* 71, **82**,*195, 196*	71, **82***, 196*
Patient #62	**16**, 71, **82**	**82**
Patient #63	*14,* **16,** *19, 20,21,* 71, **82**	**16,** *14*
Patient #64	6, *20, 21, 25,* 71, **82**, *195*	6, **16**
Patient #65	**16,** 71	None
Patient #66	**16**, 71, **82**	None
Patient #67	6, **16**	None
Patient #68	**16**, *20,* 71	None
Patient #69	**16**, 71, **82**	None
Patient #70	**16**, 71, **82**	None

Patient #71	**16,** 43, 71, **82,** 84, 86, 87, 91, *114*	43, 82*, 114*
Patient #72	6, 7, **16**	7, **16**
Patient #73	**16**	**16**
Patient #74	**16**	**16**
Patient #75	**16, 85**	**16**
Patient #76	**16**, 71, **82**	**16, 82**
Patient #77	71, **82**	**82**
Patient #78	**16**, 71, **82**	None
Patient #79	**16**, 71, **82**	None
Patient #80	**16,** *19,* **39, 68, 70,** 71, **82**	**70**
Patient #81	6, **16**	**16**
Patient #82	**16**	**16**
Patient #83	14, **16,** 19, 20, 21, 25, 71, **82**, 152,196	*14,* **16,** *152*
Patient #84	**16, 90**	**16, 90**
Patient #85	**16**, 71, **82**	**16**
Patient #86	**16**, 71, **82**	**16,** 71
Patient #87	*14,* **16,** *19, 20,* 71, **82**, *152, 195,196*	**16,** *196*
Patient #88	**16**, 71	**16**
Patient #89	**16**, 71, **82**	**16**
Patient #90	**16**, 71, **82**	None

†Non-bold face indicates Low-risk type; **boldface** indicates High-risk types; *italicized* indicates Undetermined risk Type.
